# Supplementary material for: sciCSR infers B cell state transition and predicts class-switch recombination dynamics using single-cell transcriptomic data
Source: Nat Methods. 2023 Nov 6;21(5):823–34. doi: 10.1038/s41592-023-02060-1 (PMC11093741; doi:10.1038/s41592-023-02060-1)
Supplement: Supplementary file 1 — Supplementary Notes 1–3, methods, Fig. 1 and Table 1. [file 41592_2023_2060_MOESM1_ESM.pdf]

nature methods

Article https://doi.org/10.1038/s41592-023-02060-1

# sciCSR infers B cell state transition and predicts class-switch recombination dynamics using single-cell transcriptomic data

In the format provided by the authors and unedited

## Table of Contents

*Supplementary Notes* ........ *2*

**Supplementary Note 1. Robustness of sciCSR predictions in systems with limited CSR activity.** .. **2**

**Supplementary Note 2. Robustness of sciCSR in studying rare populations.** ........ **3**

**Supplementary Note 3. Motivation of the sciCSR method** ........ **5**

*Supplementary Methods* ........ *8*

*Supplementary Figures* ........ *10*

**Supplementary Figure 1. Representative gating strategy example at day 6 for the IFNγ B cell culture data.** ........ **10**

*Supplementary Tables* ........ *11*

**Supplementary Table 1. Genomic coordinates of human and mouse constant region genes and their corresponding sterile transcript start sites** ........ **11**

## Supplementary Notes

### Supplementary Note 1. Robustness of sciCSR predictions in systems with limited CSR activity.

We further investigate the robustness of the sciCSR predictions of future isotype distributions, as a function of the current state. Specifically, we reason that in systems heavily dominated by one isotype, the lack of CSR signals beyond this dominant isotype would make predictions of future switches more noisy. To do so we resample cells from the Kim et al. dataset (Fig. ia), gradually saturating the system with the current isotype (e.g. IgG1) by randomly relabelling an increasing number of cells which has switched beyond the current isotype (e.g. IgA1 relabelled as IgG1, Fig. ib). We then fit transition models on the resampled datasets using sciCSR and compare the predicted isotype distribution against the isotype distribution measured at the subsequent timepoint. Expectedly, the more saturated the system with the current state, the less accurate the predicted isotype distribution. Specifically, predictions become noticeably problematic in cases where >95% of the cells are of one isotype (Fig. ic). Therefore, users should exercise caution when applying sciCSR to obtain predictive models from data where diversity of isotype in the population is thus limited.

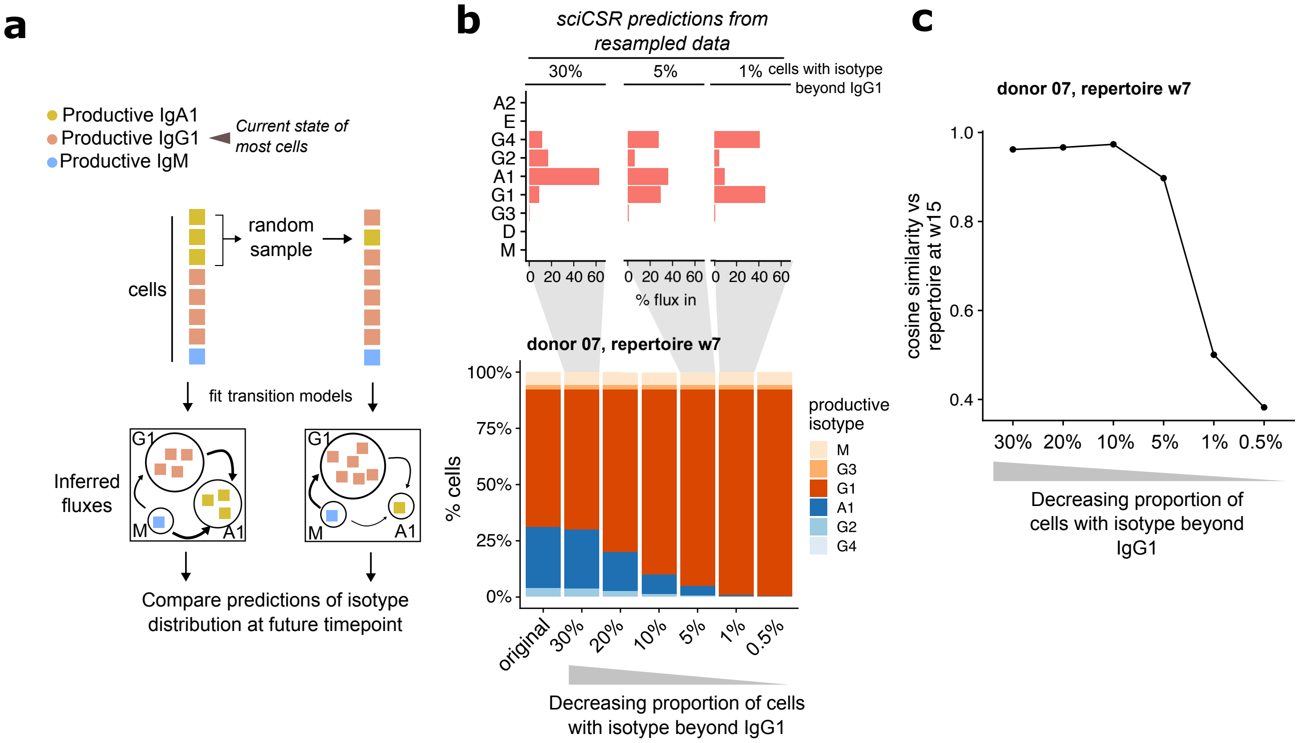


Fig. i. Robustness of sciCSR prediction in systems with limited CSR activity.

(a) Schematic to illustrate resampling of data to evaluate the impact of the current isotype predictions on sciCSR predictions of the isotype distribution at a future timepoint.

(b) Example of resampling on the donor 07, w7. The resampling generates a series of datasets (*bar plot at the bottom*) with decreasing proportion of cells which are currently beyond the dominant isotype in the distribution (IgG1 in this case). sciCSR prediction of future isotype distribution for selected resampling conditions were shown as barplots (*top*).

(c) Cosine similarity between sciCSR predictions made using the donor 07 w7 scRNA-seq data and w15 scBCR-seq isotype distribution, for the resampled datasets with different proportions of cells beyond the current isotype (IgG1).

**Supplementary Note 2. Robustness of sciCSR in studying rare populations.**

Here we investigate the minimum number of cells needed for sciCSR to accurately recover transitions underlying the data. Recall from the manuscript & Methods that sciCSR provides CSR/SHM potential scores for CellRank[1] to fit Markov State Models (MSM). Intuitively, the number of available observations would affect the ability of MSM to correctly identify states and infer transitions between them. One way to answer this question by generating a synthetic dataset of size comparable to test cases presented where sciCSR successfully recovers transitions, and apply sciCSR on this synthetic dataset. By downsampling the number of cells in each state and re-apply sciCSR on this data, we can then compare the inference results to the ground-truth.

*Description of synthetic dataset*

We consider a simple mixture of three states: IgM, IgG1 and IgA1, and the class-switching dynamics between these states. This can be taken as the transitions between three B cell subsets: Naïve, Classical Memory, DN1, as we found previously that these B cell subsets are enriched respectively in IgM/IgG1/IgA1 expression[2] (Stewart et al). For simplicity we set each state to have an equal number (*n*) of cells, and calculate CSR potential as input for sciCSR. *n* is the only variable in the simulation.

We require two inputs: (1) transcriptomic gene counts to build k-nearest neighbour (kNN) graph to describe the structure of the data, and (2) CSR/SHM potentials as pseudotime ordering to bias the kNN graph and describe the transitions. Here we used the splatter[3] package to simulate a count matrix of 2,000 genes for a three-group (or 'state') mixture with 10,000 cells in each state to constitute the ground-truth; the Stewart et al[2] dataset of healthy volunteer B cells in circulation was used as input such that the splatter-simulated gene counts would fit to a distribution similar to the Stewart et al. data. The splatter-simulated gene counts served to build the kNN graph. To assign the CSR potential, we randomly sampled the NMF-decomposed weights from Stewart et al and assign to each state, as follows:

- Cells in the IgM state were randomly assigned CSR potential of a Naïve cell from Stewart et al.;
- Cells in the IgG1 state were randomly assigned CSR potential of a C-mem cell, and;
- Cells in the IgA1 state were randomly assigned CSR potential of a DN1 cell.

As mentioned above, these cell subsets were characterised by enrichment of these isotypes in scRNA-seq data[2]. sciCSR was invoked with the CSR potential and the kNN graph as inputs with default parameters. The resulting fluxes were compared against the ground-truth. In this simulation, with an equal number of cells in each state we would expect that the majority (~ 50%) of the total flux to be attributed to transitions from IgM to IgA1 directly, and the remaining flux separated equally between IgM → IgG1 and IgG1 → IgA1. Fig. ii below demonstrates that sciCSR inference recapitulates this expected outcome, and the inference is robust across a range of cell population sizes, up to groups of *n* = 10 cells.

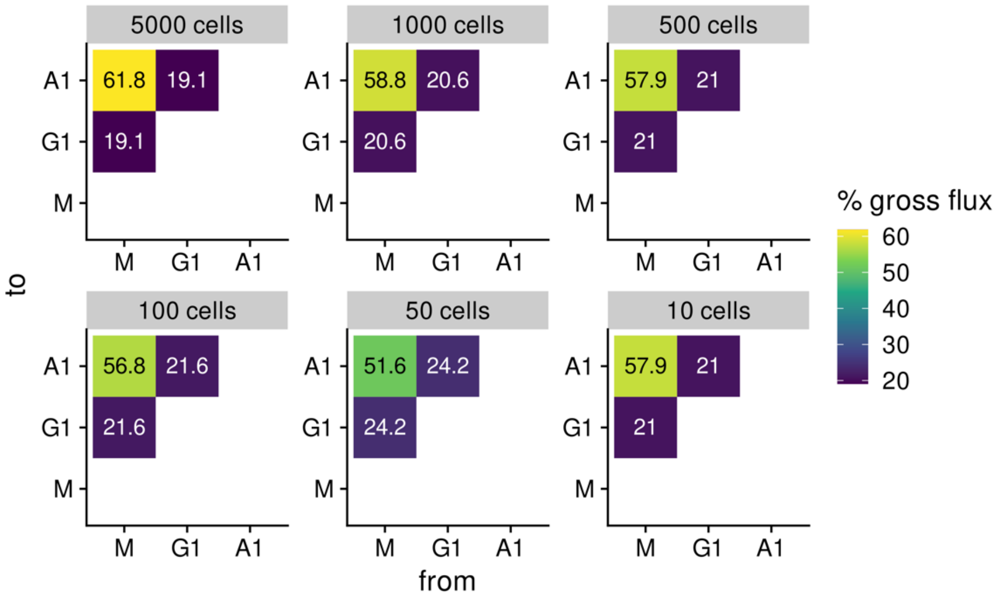


Fig. ii. sciCSR inference on a simulated mixture of IgM, IgG1 and IgA1 cells, with equal numbers of cells belonging to each isotype. Data were downsampled to the stated number of cells per group before inference using sciCSR. Heat colour corresponds to the proportion of flux attributed to each transition.

**References**


1. Lange, M. *et al.* CellRank for directed single-cell fate mapping. *Nat. Methods* **19**, 159–170 (2022).
2. Stewart, A. *et al.* Single-Cell Transcriptomic Analyses Define Distinct Peripheral B Cell Subsets and Discrete Development Pathways. *Front. Immunol.* **12**, 602539 (2021).
3. Zappia, L., Phipson, B. & Oshlack, A. Splatter: simulation of single-cell RNA sequencing data. *Genome Biol.* **18**, 174 (2017).

**Supplementary Note 3. Motivation of the sciCSR method**

*A. Challenges to model and predict class-switch recombination (CSR) dynamics in scRNA-seq data*

In recent years there have been attempts to build predictive models of class-switch recombination (CSR) in B cells. For example, based on a lineage-tracing system, Horton et al. predicts the CSR fate of a given B cell using (i) sterile transcription level and (ii) AID (Aicda) transcription level of predecessor cells of the same lineage. Here in building predictive models based on scRNA-seq data there are a number of complicating factors:

1. In scRNA-seq datasets, AID transcription expression is typically very low, and is observed almost exclusively in GC B cells. Whilst this is expected, it renders a CSR predictive model based on this feature contingent on the read depth of the scRNA-seq libraries and the cell type makeup of the sample.
2. Many scRNA-seq library preparation protocols, including droplet-based and cell-sorting-based methods, typically sample a very limited number of B cells which preclude the definition of B cell lineages where CSR can be followed through time. Therefore, while we typically could sample thousands of single B cells in a scRNA-seq (and scBCR-seq in parallel) experiment, we would be analysing a mosaic of many B cell lineages, each the result of a very sparse sampling regime of B cells at variable stages of their CSR trajectories.

Point (2) above necessitates compromises to be made in devising methods to model CSR dynamics: whilst it is very likely that the vast majority of sampled cells in the scRNA-seq data will be part of small clonotypes, in most cases the experimental design implies that this heterogeneous mixture of lineages is subject to the same condition (immune challenge/stimuli etc.) and therefore would exhibit commonalities in their behaviour to class-switch. Therefore the idea here is to consider the dynamics of groups of B cells: by considering the class-switch behaviour of a heterogeneous group of B cell lineages as a whole, sciCSR attempts to offer a description of the CSR dynamics across these lineages. The single-cell resolution of the underlying data allows us to capture the diversity within the population and account for this in the models. We feel that this represents an approach which is broadly applicable to an ever-growing spectrum of publicly available B cell scRNA-seq datasets.

We next define the "CSR prediction" problem. Specifically, we need a way to define the 'groups' of B cells for which the dynamics of transitions between these groups are to be described, as well as the sources of information with which the likelihood of transitions can be estimated:

- For grouping cells to predict CSR, the natural choice is to use productive transcript expression to group the cells. Since the definition of productive transcripts would require at least 2 reads in scRNA-seq data (1 mapping to the VDJ gene segment, 1 mapping to the coding exon for the C region) and therefore impose additional sparsity (see Supplementary Figure S3), we use the scBCR-seq data to annotate the productive isotype of each B cell, wherever such data is available. This takes advantage of the targeted enrichment of productive transcripts in the generation of scBCR-seq libraries.
- For instructing transitions, we have shown in the main text the CSR potential (Figure 4), derived using both productive and sterile transcript expression patterns from the scRNA-seq data, is able to order B cells by their maturation stages. This score can be used effectively as a pseudotemporal ordering to order the B cells and construct a transition matrix that describes the transition likelihood between single B cells.

*B. Markov models and Transition Path Theory*

**CSR formulated as Markov models**: Following from (A), the problem of predicting CSR is operationally defined as modelling the transitions between groups of B cells of each given productive isotype. We approach this by constructing a Markov chain between these isotypes, using the transition matrix constructed using CSR potential. (Alternatively, we can also group cells differently, and/or use somatic hypermutation [SHM] instead to derive the transition matrix.) These Markov chains are just like those typical of any other applications of Markovian methods: we can, for example, use the sciCSR Markov chains to simulate new sequences of states (switching events) which are consistent with the seen data generated under the same experimental condition (we used this property to compare the transition information embedded in CSR/SHM/RNA velocity, by comparing the frequency different cell states are visited in these simulated sequences, see main text Figure 6d-f).

**Differences versus conventional Markov models**: Classically, in applying Markov chains to model biological sequences and phylogenies, we are interested in a maximum likelihood solution to describe the observed sequences and/or tree topologies. Whilst we can formulate the problem of modelling CSR as nominating a maximum-likelihood solution, i.e. the most likely series of switching events that is consistent with the given data, here we are interested in the dynamics of these switching events, not only the maximum-likelihood solution: given the data, we aim to extract all the possible transitions and describe their dynamics, assigning likelihood of sampling these transitions in the Markov chains. Note this is also in contrast to models of cellular dynamics in developmental biology systems, where there is normally one clear pathway towards a certain

cell fate from the progenitor; here we are interested more in the range of possible pathways the system can take in CSR.

Transition Path Theory (TPT) provides a solution which allows us to analyse this ensemble of transition paths: we start at a pre-defined source state and enumerate, given the transition likelihoods between the states, possible paths which can traverse the states and reach the (again predefined) target. This allows us to calculate the frequency of transition paths that would flow between any two states: this represents the "flux" depicted in the figures as output of the sciCSR method. Because TPT considers all transitions, fluxes refer to any switching events involving the two states: for example, to switch from isotype X to Y, one can switch directly without an intermediate isotype, or switch via an intermediate Z. Moreover, the transition does not always occur successfully – it is possible to have attempted transitions which ultimately fall back to the starting state because, for example, the sterile transcription level is insufficient or AID is absent. Fluxes incorporate all successful transitions that switch from state X to Y; these 'fluxes' therefore represent a holistic, information-rich summary of the dynamics of the system, normally omitted in maximum-likelihood analyses of outputs from Markov models.

## Supplementary Methods

*Analysis of simulated productive and sterile IgH transcripts*. The simulated reads (see heading "Validating workflow to identify productive and sterile IgH transcripts" in Methods) were subjected to alignment to only the chromosome 14 genomic sequence from the GRCh38 reference genome, using either HISAT21 (v2.2.1) with default parameters, or STAR2 (v2.5.1.b) using the following parameters: "--outSAMmultNmax -1 --readNameSeparator space --outSAMunmapped Within KeepPairs". The resultant BAM files were then subject to productive/sterile transcript enumeration using the pipeline described above. We attempted in parallel quantification using a pseudoalignment tool for validation; salmon3 (v1.9.0) was used by supplying a custom genome containing the GRCh38 chromosome 14 genomic sequence and all reference human IgH productive/sterile transcript set described above. We found that quantification by salmon was noticeably more inaccurate compared to those based on applying our pipeline to HISAT2/STAR-generated alignments. They were therefore not included in the results shown in Figure 2.

*Combining scRNA-seq and scBCR-seq data*. sciCSR provides functionalities to merge scRNA-seq and scBCR-seq measurements obtained in parallel for the same set of cells using the 10X Genomics technology. sciCSR expects the 10X scRNA-seq and scBCR-seq data be pre-processed (e.g. by using the 10X cellranger software suite), and the resultant scRNA-seq gene count matrix be imported in R and stored as a Seurat data object. We added, as separate columns in the metadata slot of the Seurat data object, sequence annotations in the filtered contigs comma-separated value (CSV) files (i.e. those with filenames "filtered_contig_annotations.csv") from the "cellranger vdj" run. The filtered contig annotations were cleaned for cases where more than one heavy and/or light chain sequence was associated to the same cell, taking only the sequence with maximum unique molecular identifier (UMI) count as representative. This data table is organised such that sequences are organised into one cell per row, listing separate heavy and light chain annotations as columns. By default the following annotations are retained and merged into the Seurat object metadata: V, D, J, C gene annotations, binary variables of whether sequences are productive and full-length, the CDR3 nucleotide and amino acid sequences, and the total read count and UMI count associated with each heavy/light chain sequence.

*Non-negative matrix factorization (NMF) analysis to derive isotype signatures*. We first compared the signature matrix *P* obtained using NMF upon incrementing the value the number of signatures *H* from 2 to *S* (i.e. the total number of isotypes), and observed that as *H* increased, we obtained more fine-grained signatures eventually ending up with signatures composed of only 1 isotype (Extended data Fig. 6a). For the human reference atlas setting *H* = 2 yields a naïve B cell signature biased towards IgM, and a memory signature which was a combination mainly of different IgG subtype productive/sterile transcripts (Extended data Fig. 6a). For the mouse atlas setting *H* = 2 did not yield separate IgM+ and IgM- signatures, but for *H* = 3 we obtained an $IgM_{sterile}$+, an $IgM_{productive}$+, and an IgG-biased signature (Extended data Fig. 6b). We therefore

directly took the human $P_{H=2}$ matrix as the reference human signature matrix, and for mouse we sum over the $IgM_{sterile}$+ and $IgM_{productive}$+ signature weights to generate a two-signature (IgM-biased, IgG-biased) $P$ matrix as the reference mouse signature matrix.

## References


1. Kim, D., Paggi, J. M., Park, C., Bennett, C. & Salzberg, S. L. Graph-based genome alignment and genotyping with HISAT2 and HISAT-genotype. *Nat. Biotechnol.* **37**, 907–915 (2019).
2. Dobin, A. et al. STAR: ultrafast universal RNA-seq aligner. *Bioinformatics* **29**, 15–21 (2013).
3. Patro, R., Duggal, G., Love, M. I., Irizarry, R. A. & Kingsford, C. Salmon provides fast and bias-aware quantification of transcript expression. *Nat. Methods* **14**, 417–419 (2017).

## Supplementary Figures

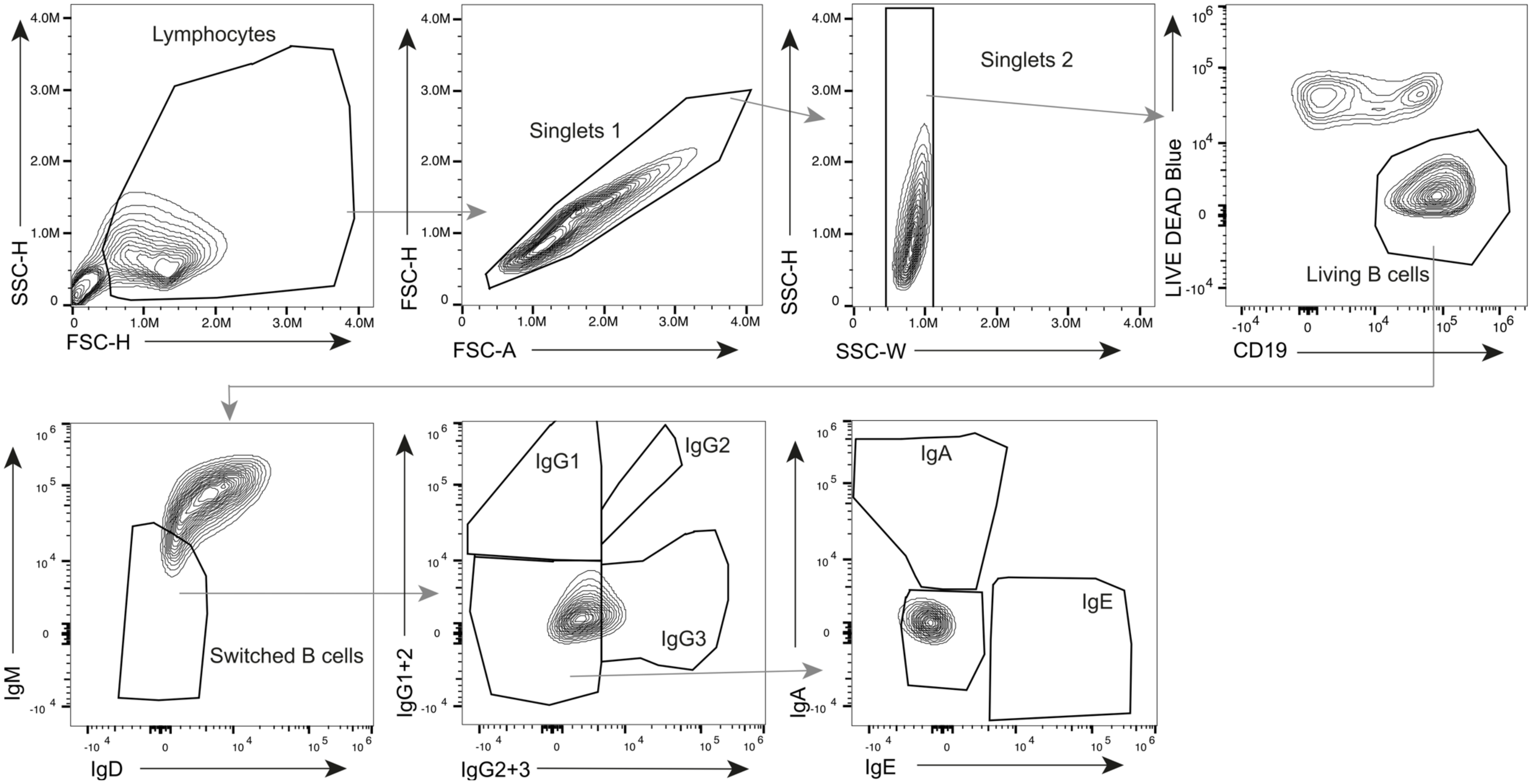


**Supplementary Figure 1. Representative gating strategy example at day 6 for the IFNγ B cell culture data.**

In order to identify class-switched B cells after culture, lymphocytes, singlets and living B cell were gated. From living B cells, switched B cells were identified as $IgM^-$ and $IgD^-$. Within switched B cells, $IgG1^+$, $IgG2^+$, $IgG3^+$, $IgA^+$ and $IgE^+$ B cells were identified.

## Supplementary Tables

**Supplementary Table 1. Genomic coordinates of human and mouse constant region genes and their corresponding sterile transcript start sites.** Sterile transcript start sites were mapped by aligning collected sterile transcript sequences from the literature to the hg38 (human) and mm10 (mouse) reference genome assemblies (see Methods). Note that the IgH genes are located on the minus strand for both mouse and human. This table is shipped with the sciCSR R package.

| Ensembl gene ID | Gene | Genomic coordinate | Sterile transcript start site | Genome assembly | Organism |
|---|---|---|---|---|---|
| ENSG00000211899 | *IGHM* | chr14:105,851,705-105,856,218 | 105,861,958 | hg38 | Human |
| ENSG00000211897 | *IGHG3* | chr14:105,764,503-105,771,405 | 105,775,495 | hg38 | Human |
| ENSG00000211896 | *IGHG1* | chr14:105,736,343-105,743,071 | 105,748,177 | hg38 | Human |
| ENSG00000211895 | *IGHA1* | chr14:105,703,995-105,708,665 | 105,712,867 | hg38 | Human |
| ENSG00000211893 | *IGHG2* | chr14:105,639,559-105,644,790 | 105,648,651 | hg38 | Human |
| ENSG00000211892 | *IGHG4* | chr14:105,620,506-105,626,066 | 105,629,851 | hg38 | Human |
| ENSG00000211891 | *IGHE* | chr14:105,597,691-105,601,728 | 105,605,176 | hg38 | Human |
| ENSG00000211890 | *IGHA2* | chr14:105,583,731-105,588,395 | 105,591,896 | hg38 | Human |
| ENSMUSG00000076617 | *Ighm* | chr12:113,418,558-113,422,730 | 113,427,389 | mm10 | Mouse |
| ENSMUSG00000076615 | *Ighg3* | chr12:113,356,224-113,361,232 | 113,366,394 | mm10 | Mouse |
| ENSMUSG00000076614 | *Ighg1* | chr12:113,325,240-113,330,523 | 113,339,379 | mm10 | Mouse |
| ENSMUSG00000076613 | *Ighg2b* | chr12:113,302,965-113,307,933 | 113,314,819 | mm10 | Mouse |
| ENSMUSG00000076612 | *Ighg2c* | chr12:113,285,325-113,288,932 | 113,296,262 | mm10 | Mouse |
| ENSMUSG00000087642 | *Ighe* | chr12:113,269,260-113,273,248 | 113,277,549 | mm10 | Mouse |
| ENSMUSG00000095079 | *Igha* | chr12:113,254,830-113,260,236 | 113,265,138 | mm10 | Mouse |
