# Supplementary material for: sciCSR infers B cell state transition and predicts class-switch recombination dynamics using single-cell transcriptomic data
Source: Nat Methods. 2023 Nov 6;21(5):823–34. doi: 10.1038/s41592-023-02060-1 (PMC11093741; doi:10.1038/s41592-023-02060-1)
Supplement: Supplementary file 2 — Reporting Summary [file 41592_2023_2060_MOESM2_ESM.pdf]

Corresponding author(s): Prof. Franca Fraternali, Dr.Joseph Ng

Last updated by author(s): Aug 17, 2023

Reporting Summary

Nature Portfolio wishes to improve the reproducibility of the work that we publish. This form provides structure for consistency and transparency in reporting. For further information on Nature Portfolio policies, see our Editorial Policies and the Editorial Policy Checklist.

Statistics

For all statistical analyses, confirm that the following items are present in the figure legend, table legend, main text, or Methods section.

n/a	Confirmed	
☐	☒	The exact sample size (*n*) for each experimental group/condition, given as a discrete number and unit of measurement
☐	☒	A statement on whether measurements were taken from distinct samples or whether the same sample was measured repeatedly
☐	☒	The statistical test(s) used AND whether they are one- or two-sided<br>*Only common tests should be described solely by name; describe more complex techniques in the Methods section.*
☐	☒	A description of all covariates tested
☐	☒	A description of any assumptions or corrections, such as tests of normality and adjustment for multiple comparisons
☐	☒	A full description of the statistical parameters including central tendency (e.g. means) or other basic estimates (e.g. regression coefficient) AND variation (e.g. standard deviation) or associated estimates of uncertainty (e.g. confidence intervals)
☐	☒	For null hypothesis testing, the test statistic (e.g. *F*, *t*, *r*) with confidence intervals, effect sizes, degrees of freedom and *P* value noted<br>*Give P values as exact values whenever suitable.*
☒	☐	For Bayesian analysis, information on the choice of priors and Markov chain Monte Carlo settings
☐	☒	For hierarchical and complex designs, identification of the appropriate level for tests and full reporting of outcomes
☐	☒	Estimates of effect sizes (e.g. Cohen's *d*, Pearson's *r*), indicating how they were calculated

Our web collection on statistics for biologists contains articles on many of the points above.

Software and code

Policy information about availability of computer code

Data collection	We used publicly available data deposited in the Gene Expression Omnibus (GEO) and ArrayExpress. FASTQ files were downloaded either by using the wget command-line utility (for ArrayExpress) or the NCBI SRA Toolkit (v2.11.1) (for GEO)
Data analysis	Code for data analysis presented in this manuscript has been implemented in the R package sciCSR which is available at: https://github.com/Fraternalilab/sciCSR. Documentation and vignettes can be found in the GitHub repository. Analysis notebooks and code used in generating the analysis presented in this manuscript can be found at https://github.com/Fraternalilab/sciCSR-analysis. Analysis presented in this manuscript was performed using R version v4.2.2. For the simulated dataset (Fig. 2), they were generated and analysed using the R package polyester (v1.29.1.1), as well as command-line programs HISAT2 (v2.2.1) and STAR (v2.5.1.b).

For manuscripts utilizing custom algorithms or software that are central to the research but not yet described in published literature, software must be made available to editors and reviewers. We strongly encourage code deposition in a community repository (e.g. GitHub). See the Nature Portfolio guidelines for submitting code & software for further information.

Data

Policy information about availability of data

All manuscripts must include a data availability statement. This statement should provide the following information, where applicable:

- Accession codes, unique identifiers, or web links for publicly available datasets
- A description of any restrictions on data availability
- For clinical datasets or third party data, please ensure that the statement adheres to our policy

The scRNA-seq and scBCR-seq data of the IFNγ culture experiment are accessible via ArrayExpress (accession E-MTAB-13050). All other datasets used in this work are publicly available: Kim et al.78 (Gene Expression Omnibus [GEO] entry GSE195673), Gómez-Escolar et al.79 (GSE189775), Hong et al.80 (GSE145922), Stewart et al.10 (E-MTAB-9544), King et al.11 (E-MTAB-9005), Mathew et al.13 (E-MTAB-9478 and E-MTAB-9491) and Luo et al.14 (E-MTAB-10081). For GEO entries raw FASTQ files were downloaded from the associated Sequence Read Archive (SRA) entries. Processed data files generated in this study can be found in the Zenodo repository https://dx.doi.org/10.5281/zenodo.8005705. Reference genome data (hg38, mm10) used in aligning scRNA-seq datasets were obtained from the 10x cellranger website (https://support.10xgenomics.com/single-cell-vdj/software/downloads/latest).

Human research participants

Policy information about studies involving human research participants and Sex and Gender in Research.

Reporting on sex and gender	For the in vitro experiments, they were performed using PBMCs from 3 healthy controls. Although sex is not know to have an effect on the readouts shown in this publication, we aimed to balance this factor by choosing 2 males and one female.
Population characteristics	Participants were reported as healthy. They specifically reported free of immune-related diseases. Age was restricted between 25 and 45 years of age as this factor is known to affect immune-related readouts. Ethnicity of the participants/ healthy controls was reported as caucasian.
Recruitment	Healthy adults were recruited as part of an effort of the research team to build a repository of healthy controls. Samples from this repository include research team co-workers and public health care workers. No self-selection bias that could impact the results has been identified.
Ethics oversight	Ethical approval was obtained from the College London Hospital (UCLH) Health Service ethical committee, under REC reference no. 14/SC/1200. Informed consent was obtained from all donors.

Note that full information on the approval of the study protocol must also be provided in the manuscript.

Field-specific reporting

Please select the one below that is the best fit for your research. If you are not sure, read the appropriate sections before making your selection.

☒ Life sciences ☐ Behavioural & social sciences ☐ Ecological, evolutionary & environmental sciences

For a reference copy of the document with all sections, see nature.com/documents/nr-reporting-summary-flat.pdf

Life sciences study design

All studies must disclose on these points even when the disclosure is negative.

Sample size	For the in vitro experiments, sample size was chosen based on preliminary experiments where results of IgG induction were consistent across all donors and meaningful differences between groups were achieved. scRNA-seq data were collected from three donors to ensure data cover sufficient cells and also that the data analysed cover both male and female. Analysis was performed on biological samples for which scRNA-seq and scBCR-seq (i.e. VDJ B-cell receptor sequencing) data were publicly available and complete.
Data exclusions	All biological samples from the cited reports for which complete scRNA-seq and scBCR-seq data are publicly available have been included in the analysis. Similarly, all data from in vitro experiments has been included.
Replication	Analysis can be replicated by using the sciCSR R package made available as part of this work. The algorithm does not involve random initialisation and therefore results are consistent across multiple runs. In vitro experiments using the same conditions as the one featured in this publication have been reproduced at least 3 times with similar results.
Randomization	Not applicable, since all biological samples have been labelled by their biological identities/genotypes etc. in the relevant entries in data repositories. For the in vitro experiments, randomization is not applicable since this study did not aim to discover the effect of a specific treatment condition, but rather as a time-course study to monitor IgG induction. Cells from all three donors were subject to the same time-course.
Blinding	Not applicable, since all biological samples have been labelled by their biological identities/genotypes etc. in the relevant entries in data repositories. Similarly, sequencing data from in vitro experiment was labelled with relevant biological condition.

Reporting for specific materials, systems and methods

We require information from authors about some types of materials, experimental systems and methods used in many studies. Here, indicate whether each material, system or method listed is relevant to your study. If you are not sure if a list item applies to your research, read the appropriate section before selecting a response.

Materials & experimental systems

n/a	Involved in the study
☐	☒ Antibodies
☒	☐ Eukaryotic cell lines
☒	☐ Palaeontology and archaeology
☒	☐ Animals and other organisms
☒	☐ Clinical data
☒	☐ Dual use research of concern

Methods

n/a	Involved in the study
☒	☐ ChIP-seq
☐	☒ Flow cytometry
☒	☐ MRI-based neuroimaging

Antibodies

Antibodies used

Anti-human CD19 - BV785 (Biolegend, Cat# 302240, clone HIB19, lot# B339489, dilution 1:200)
Anti-human CD27 - BV711 (BD, Cat# 740291, clone M-T271, lot# 2140520, dilution 1:200)
Anti- human CD24 - PE-Cy7 (Biolegend, Cat# 311120, clone ML5, lot# B5345471, dilution 1:200)
Anti-human CD38 - BV605 (Biolegend, Cat# 356642, clone HB-7, lot# B310479, dilution 1:200)
Anti-human IgD - PerCP-Cy5.5 (BD, Cat# 561315, clone IA6-2, lot# 1060319, dilution 1:200)
Anti-human IgM - APC/Fire™ 750 (Biolegend, Cat# 314546, clone MHM-88 , lot# B283355, dilution 1:200)
Anti-human IgG1 - PE (Cytogonos, Cat# CYT-IGG1PE , clone SAG1, lot# 2207125, dilution 1:200)
Anti-human IgG2 - PE (Cytogonos, Cat# CYT-IGG2PE , clone SAG2, lot# 2112026, dilution 1:200)
Anti-human IgG2 - FITC (Cytogonos, Cat# CYT-IGG2F , clone SAG2, lot# 2112024/2, dilution 1:200)
Anti-human IgG3 - FITC (Cytogonos, Cat# CYT-IGG3F , clone SAG3, lot# 2207060, dilution 1:200)
Anti-human IgA - PE-Vio 615 (Miltenyi Biotec, Cat# 130-116-882 , clone REA1014, lot# 5220507430, dilution 1:200)
Anti-human IgE - BUV615 (BD, Cat# 751346, clone G7-28, lot# 3046667, dilution 1:200)

Validation

All antibodies has been validated by other publications and/or the manufacturer in the format used in the present study. In addition, all antibodies were validated in total PBMCs as positive control where clear positive and negative populations were observed.

Flow Cytometry

Plots

Confirm that:

☒ The axis labels state the marker and fluorochrome used (e.g. CD4-FITC).

☒ The axis scales are clearly visible. Include numbers along axes only for bottom left plot of group (a 'group' is an analysis of identical markers).

☒ All plots are contour plots with outliers or pseudocolor plots.

☒ A numerical value for number of cells or percentage (with statistics) is provided.

Methodology

Sample preparation

Cultured B cells in IgG polarising class-switch medium from each donor were collected and stained for subsequent flow cytometry measurement. Anti-CD19, anti-CD27, anti-CD24 and anti-CD38 were used to stain the cells extracellularly. Viability staining using LIVE/DEAD™ Fixable Blue Dead Cell Stain Kit (Invitrogen, #L23105) was performed together with the surface staining. Cells were then fixed and permeabilised using the eBioscience™ Intracellular Fixation & Permeabilization Buffer Set (Invitrogen, #88-8824-00) and stained intracellularly with anti-IgD, anti-IgM, anti-IgG1, anti-IgG2, anti-IgG3, anti-IgA and anti-IgE.

Instrument

Sorter: BD FACSAria™ Fusion
Analyser: CytekTM Aurora cytometer (5 lasers)

Software

Acquisition software for sorter: BD FACSDiva version 9.4
Acquisition software for analyser: SpectroFlo version 3.0.3 with automated unmixing.
Analysis software: FlowJo version 10.8.1

Cell population abundance

Reported ratios in the figures are percentages of positive population among total B cells.

Gating strategy

Lymphocytes were selected based on SSC-H (0.1x10^6 to 3x10^6) and FCS-H (0.5x10^6 to 4x10^6). Doublets were excluded in two consecutive gates. First by using FCS-H (0.5x10^6 to 4x10^6) vs FCS-A (0.4x10^6 to 4x10^6) and selecting the singlets

found in the diagonal. Then a second time using SSC-H (0.1x10^6 to 4x10^6) and SSC-W (<0.2 x10^6). Once doublets were excluded, living B cells were selected as CD19 positive (>10^4) cells and negative for the viability dye (<10^4). From living B cells, double-negative cells for IgM (<2x10^4) and IgD (<10^3) were selected as class-switched B cells. Within class-switched B cells, IgG1, IgG2 or IgG3 positive cells were selected based on a combination of antibodies (anti-IgG1 PE, anti-IgG2 PE, anti-IgG2 FITC and anti-IgG3 FITC). For this gating FITC positivity was considered at >10^3, and PE positivity was considered at >10^4. Withing class-switched B cells without expression of IgG1/2/3, IgA (>10^3) or IgE (>10^3) positive B cell were gated. Gating strategy for immunophenotyping of cultured B cells are presented in Supplementary Figure 1.

☒ Tick this box to confirm that a figure exemplifying the gating strategy is provided in the Supplementary Information.
